# Basal Gene Expression by Lung CD4+ T Cells in Chronic Obstructive Pulmonary Disease Identifies Independent Molecular Correlates of Airflow Obstruction and Emphysema Extent

**DOI:** 10.1371/journal.pone.0096421

**Published:** 2014-05-07

**Authors:** Christine M. Freeman, Alexandra L. McCubbrey, Sean Crudgington, Joshua Nelson, Fernando J. Martinez, MeiLan K. Han, George R. Washko, Stephen W. Chensue, Douglas A. Arenberg, Catherine A. Meldrum, Lisa McCloskey, Jeffrey L. Curtis

**Affiliations:** 1 Research Service, VA Ann Arbor Healthcare System, Ann Arbor, Michigan, United States of America; 2 Pulmonary and Critical Care Medicine Division, Department of Internal Medicine, University of Michigan Health System, Ann Arbor, Michigan, United States of America; 3 Graduate Program in Immunology, University of Michigan, Ann Arbor, Michigan, United States of America; 4 Division of Pulmonary and Critical Care Medicine, Department of Medicine, Brigham & Womans Hospital and Harvard University, Boston, Massachusetts, United States of America; 5 Pathology and Laboratory Medicine Service, VA Ann Arbor Healthcare System, Ann Arbor, Michigan, United States of America; 6 Department of Pathology, University of Michigan Health System, Ann Arbor, Michigan, United States of America; 7 Pulmonary and Critical Care Medicine Section, Medicine Service, VA Ann Arbor Healthcare System, Ann Arbor, Michigan, United States of America; University of Alabama-Birmingham, United States of America

## Abstract

Lung CD4+ T cells accumulate as chronic obstructive pulmonary disease (COPD) progresses, but their role in pathogenesis remains controversial. To address this controversy, we studied lung tissue from 53 subjects undergoing clinically-indicated resections, lung volume reduction, or transplant. Viable single-cell suspensions were analyzed by flow cytometry or underwent CD4+ T cell isolation, followed either by stimulation with anti-CD3 and cytokine/chemokine measurement, or by real-time PCR analysis. In lung CD4+ T cells of most COPD subjects, relative to lung CD4+ T cells in smokers with normal spirometry: (a) stimulation induced minimal IFN-γ or other inflammatory mediators, but many subjects produced more CCL2; (b) the T effector memory subset was less uniformly predominant, without correlation with decreased IFN-γ production. Analysis of unstimulated lung CD4+ T cells of all subjects identified a molecular phenotype, mainly in COPD, characterized by markedly reduced mRNA transcripts for the transcription factors controlling T_H_1, T_H_2, T_H_17 and FOXP3+ T regulatory subsets and their signature cytokines. This mRNA-defined CD4+ T cell phenotype did not result from global inability to elaborate mRNA; increased transcripts for inhibitory CD28 family members or markers of anergy; or reduced telomerase length. As a group, these subjects had significantly worse spirometry, but not DLCO, relative to subjects whose lung CD4+ T cells expressed a variety of transcripts. Analysis of mRNA transcripts of unstimulated lung CD4+ T cell among all subjects identified two distinct molecular correlates of classical COPD clinical phenotypes: basal IL-10 transcripts correlated independently and inversely with emphysema extent (but not spirometry); by contrast, unstimulated IFN-γ transcripts correlated independently and inversely with reduced spirometry (but not reduced DLCO or emphysema extent). Aberrant lung CD4+ T cells polarization appears to be common in advanced COPD, but also exists in some smokers with normal spirometry, and may contribute to development and progression of specific COPD phenotypes.

**Trial Registration:**

ClinicalTrials.gov as NCT00281229

## Introduction

Chronic obstructive pulmonary disease (COPD) is a growing cause of worldwide death and disability [1]. COPD is an inflammatory condition [2] triggered by inhaled oxidative stress, most commonly from cigarette smoking or indoor air pollution [3]. COPD is characterized by airflow limitation resulting from alveolar destruction plus airway changes (including loss of elasticity, wall thickening and increased mucus production). Once initiated, this airflow limitation is incompletely reversible and progressive despite removal of the inciting stress, as by smoking cessation [4]. Because no current medications halt COPD progression or alter mortality, improved understanding of its pathogenesis is crucial.

As COPD progresses, multiple leukocyte types accumulate in the lungs [5]. Best studied have been lung CD8+ T cells, which increase production of IFN-γ [6]–[8] and cytotoxic molecule expression [9] in direct correlation with spirometrically-defined COPD severity. Lung CD4+ T cells have been less well-explored, although their numbers also steadily increase [5], [10], [11]. Several further findings imply that lung CD4+ T cells might contribute to COPD progression. Advanced COPD is associated with appearance in the distal lung parenchyma of lymphoid follicles containing germinal centers [5], which logically depend on T cell help. CD4+ T cells from emphysematous lungs showed reduced T cell receptor (TCR) diversity implying oligoclonality and were more readily expanded in vitro by IL-2, relative to those of never-smokers without lung disease [12]. Expression of CD69, a cell-surface receptor traditionally considered to be an early and transient activation marker, by lung CD4+ T cells (and lung CD8+ T cells) correlated both with spirometrically-defined COPD severity and with the expression of co-stimulatory molecules on lung dendritic cells (DC) from the same individuals [13]. Immunohistochemical analysis demonstrated close contact between CD4+ T cells and DC in the lung parenchyma [13]. Lung CD4+ T cells have been implicated in organ-specific autoimmune lung destruction [14]. CD4+ T cells polarized to T_H_1 and T_H_17 phenotypes have been identified in the lungs of emphysema patients, but not in lungs of control subjects [15]. Collectively, these findings imply that lung CD4+ T cells are acutely activated in advanced COPD, but their precise function in pathogenesis remains controversial. Hence, better understanding of the functional capacities of lung CD4+ T cells is an important intermediate goal in development of novel therapies to limit COPD progression.

In the current study, we used in vitro stimulation, flow cytometry and real-time PCR to analyze human lung CD4+ T cells from lung tissue resected for clinical indications. The results were unexpected, based on the Tc1 effector-memory phenotype that we previously found for lung CD8+ T cells (in many cases from the same subjects as used in this study). Instead, despite a decided predominance of the T effector-memory (T_EM_) phenotype, lung CD4+ T cells from many COPD subjects produced CCL2 but virtually no IFN-γ or other inflammatory cytokines following TCR stimulation, relative to smokers with preserved lung function. Hierarchical clustering analysis of unstimulated RNA transcripts from isolated lung CD4+ T cells revealed that a subset of subjects, mostly with COPD, were virtually devoid of transcripts for T_H_1, T_H_2, T_H_17, and T regulatory (T_Reg_) transcription factors and cytokines, although they expressed some upregulated transcripts that argued against global shutdown of RNA synthesis. Further analysis identified associations of deficient IL-10 with emphysema versus IFN-γ with airflow obstruction without emphysema. These cross-sectional data suggest that independent impairments in the ability of lung CD4+ T cell to produce specific cytokines might contribute to progression of specific disease phenotypes in some susceptible smokers.

## Methods and Materials

### Ethics Statement

Studies and consent procedures were performed in accordance with the Declaration of Helsinki at the VA Ann Arbor Healthcare System and the University of Michigan Health System and were approved by the Institutional Review Board at each site (FWA 00000348 and FWA 00004969, respectively). Written consent to participate in the study was obtained preoperatively. The institutional review boards have examined the protocols and certified that “The risks are reasonable in relation to benefits to subjects and the knowledge to be gained. The risks of the study have been minimized to the extent possible.”

### Subject Enrollment & Patient Populations

Lung tissue was obtained from consented subjects undergoing clinically-indicated resections for pulmonary nodules, lung volume reduction surgery, or lung transplantation. All subjects (*n* = 53) underwent preoperative spirometry, collection of a variety of clinical data including medications and history of recent (6 weeks) respiratory infections, and clinical evaluation by a pulmonologist. When available, non-contrast-enhanced CT scans (*n* = 17) were analyzed for percent emphysema using 3D Slicer software (www.airwayinspector.org) and a threshold of <−950 Hounsfield units. Data on lung CD8+ T cells and lung dendritic cells on some subjects in this study have been described previously [9], [13].

We categorized subjects using the 2008 classification of the Global Initiative for Chronic Obstructive Lung Disease (GOLD) [16]. Subjects (*n* = 16) with a history of smoking >10 pack years, a ratio of forced expiratory volume in 1 second to forced vital capacity (FEV_1_/FVC) >0.70, normal spirometry, and no clinical diagnosis of COPD represent smoking controls. Subjects (*n* = 37) with a smoking history >10 pack years, FEV_1_/FVC <0.7 and abnormal spirometry were considered to have COPD. Table S1 shows the number of subjects, sex ratio, age, smoking history, spirometry, emphysema percent, and inhaled corticosteroid (ICS) use for the entire cohort. Due to limitations in sample size and cell yield, not all experiments could be performed on all subjects, and conversely, some subjects were used in more than one type of experiment.

### Sample Preparation & Experimental Design

We collected only non-neoplastic lung tissue remote from any nodules and lacking post-obstructive changes as determined by a pathologist. Lung tissue (∼3 g) was mechanically disaggregated without enzymes, which we have previously shown yields a single cell suspension of high viability and functional competence as assayed in vitro [8], [13], [17]. Cells were filtered through a 40 µm strainer to remove debris, then were used in one or more ways: immediate processing for flow cytometry (*n* = 22); or isolation of CD4+ T cells. To isolate lung CD4+ T cells, unlabelled lung homogenate was first passed through a Macs LS column (Miltenyi Biotec, Auburn, CA) removing large cells, especially macrophages, which remain stuck in the column. Next, human-specific CD4 microbeads and positive selection on second Macs LS columns further enriched for CD4+ cells [9], which were used for real-time PCR analysis of RNA (*n* = 31) or in vitro experiments (*n* = 29).

### Flow Cytometric Analysis

Cells were stained as previously described [13]. We used monoclonal antibodies against the following antigens (clones shown in parentheses): CD45 (HI30), CD3 (HIT3a), CD4 (OKT4), CD8 (HIT8a), CD56 (N-CAM), CD27 (O323) and CD62L (Dreg 56) (eBioscience, San Diego, CA). Antibodies and isotype-matched controls were directly conjugated to either fluorescein isothiocyanate (FITC), phycoerythrin (PE), phycoerythrin- cyanine 7 (PE-Cy7), allophycocyanin (APC), Pacific Blue, Alexa Fluor 700, or biotin, with the biotinylated antibodies developed using streptavidin-phycoerythrin-cyanine 5 (SA-PE-Cy5). We assessed viability in all experiments using a Live/Dead Fixable Near-IR Dead Cell Stain Kit for 633 nm excitation (Invitrogen, Carlsbad, CA). Immediately after staining, cells were fixed and stored in staining buffer plus 2% paraformaldehyde; tubes were then stored at 4°C in a rack wrapped in aluminum foil until analyzed.

Experiments were performed on an LSR II flow cytometer (BD Bioscience, San Jose, CA), equipped with the following lasers: 488 nm blue, 405 nm violet laser, 633 nm red HeNe laser, and a 561 nm yellow-green laser. Data were collected using FACSDiva software (BD Biosciences) with automatic compensation and were analyzed using FlowJo software (Tree Star, Ashland, OR). We collected at least 10,000 viable CD45+ events per sample in each experiment.

### CD4+ T Cell *in vitro* Stimulation

Isolated CD4+ T cells were cultured in 96-well plates at 50,000 cells per 200 µl in lymphocyte culture media (10% FBS, 1 mM sodium pyruvate, 0.5 mM 2-Mercaptoethanol, 1 mM HEPES, 100 U/mL penicillin, 100 U/ml streptomycin, 0.292 mg/mL L-Glutamine). Cells were stimulated with plate-bound anti-CD3ε (eBioscience) at a concentration of 5 µg/mL or with media alone. After 48 hours, supernatants were collected and stored at −20°C until analyzed. We determined IFN-γ, TNF-α, IL-13, IL-17A, IL-10, CCL2, CCL3, and CCL5 protein using multiplex bead sets (Invitrogen) and a Luminex 200 system (Luminex Corporation, Austin, TX), according to manufacturer’s instructions.

### Quantitative Real-time PCR

Micoro Poly(A) Pure kits (Ambion, Austin, TX) were used to isolate RNA from CD4+ T cells, and any contaminating genomic DNA was removed using DNA-free kits (Ambion). Each RNA sample was reverse-transcribed in a 20 µl reaction using SuperScript II RNase H^−^ Reverse Transcriptase (Invitrogen Corporation, Carlsbad, CA). We purchased TaqMan Universal PCR master mix and all primer-probe sets from Applied Biosystems. Transcripts were analyzed in duplicate (384-well format) on an ABI PRISM 7900HT (Applied Biosystems) using the comparative threshold cycle method, as described [9]. Delta Ct (ΔCt) was calculated by subtracting the reference gene Ct from the target gene Ct and then converted to arbitrary units (AU) with the formula: AU = 2^−ΔCt^ ×10^3^. Human glyceraldehyde-3-phosphate dehydrogenase was used as the endogenous reference gene.

### Analysis of Telomere Length by Real-time PCR

We assessed telomere length using a quantitative real-time PCR assay originally described by Cawthon [18], which we performed in duplicate using the same primers and conditions as described by Savale and colleagues [19]. This method compares copy numbers of the telomere repeat (T) to number of a single-copy gene (S) to yield a T/S ratio. The assay was performed on DNA from purified lung CD4+ T cells, which we isolated from the phenol phase and interphase of samples homogenized in TRI reagent (Ambion) following the manufacturer’s protocol. We determined concentrations of both products by the comparative threshold cycle method (T/S = 2^−ΔΔCt^) using SYBR green (Invitrogen) and a Mx3000P real-time PCR system (Stratagene). As the reference single-copy gene, we used acidic ribosomal phosphoprotein PO (36B4).

### Statistical Analyses

The majority of statistical analyses were performed using GraphPad Prism 6.0 (GraphPad Software, Inc., La Jolla, CA) on a Macintosh Quad-Core Intel Xeon computer running OS 10.8.3 (Apple; Cupertino, CA). Mann-Whitney t tests were used to compare two groups. We used nonparametric (Spearman) correlation analysis to determine the correlation coefficient, *r_S_*. Multiple linear regression was performed using SPSS Statistics 21.0 (IBM Corp.; Armonk, NY). The heat map and hierarchical clustering analyses were generated using the open-software program R 2.15.3 (http://www.r-project.org); Row Z scores were calculated from log-transformed arbitrary units (arbitrary units = 2^−(target Ct – reference Ct)^ ×10^3^). A two-tailed *p* value of <0.05 was considered to indicate significance.

## Results

### CD4+ T cells from Many COPD Subjects have Impaired Production of IFN-γ Following TCR Stimulation

To gain insight into the possible roles of lung CD4+ T cells in COPD pathogenesis, we prospectively recruited subjects (*n* = 53) undergoing clinically-indicated lung resection procedures **(** **Table 1** **)**, comparing subjects with COPD versus smokers with preserved spirometry. The groups differed significantly in smoking history expressed as pack-years, in FEV1% predicted, emphysema extent, indication for surgery and use of ICS, but not in age, sex or smoking status expressed as active versus former smokers (defined as having quit for more than 6 months). The emphysema extent found in the smokers without COPD was only slightly greater than that determined in healthy never-smokers at autopsy [20]–[22] or by comparable CT scan measurements [23].

**Table 1:** Summary of demographics, smoking history, spirometry, emphysema, and inhaled steroid usage for entire cohort^1^.

Group	Smokers with normal spirometry	COPD	*p* value
Subjects, n	16	37	
Sex ratio, M/F	11/5	25/12	0.99
Age, years (SD)	64 (11)	63 (10)	0.84
Smoking, pack-years (SD)	43 (43)	63 (36)	0.02
Smoking status (Active/Former^2^)	9/7	23/14	0.76
FEV1, % predicted (SD)	97 (14)	46 (27)	<0.0001
FEV1/FVC (SD)	0.78 (0.05)	0.45 (0.19)	<0.0001
Emphysema, % (SD)	6 (11)^2^	31 (14)	0.0006
Cancer as indication for surgery (yes/no)	16/0	18/19	0.0003
Lung transplant (yes/no)	0/16	11/26	0.02
ICS use (yes/no)	1/15	21/16	0.0006

1 Data are presented as average (SD) except for sex ratios, smoking status, indication for surgery, and ICS use; M, male; F, female; ICS, inhaled corticosteroids;

2 former smoker defined as having quit for more than six months.

Using the freshly harvested lung tissue of both groups, we performed a variety of experiments on CD4+ lung T cells. Due to limitations in tissue size and cell yield, not all experiments were performed on all individuals, and conversely, some subjects were used in more than one type of experiment. For this reason, characteristics of the subjects used in each type of experiment are shown separately in Supporting Tables S1–S6. Some of these subjects (*n* = 28) were previously described in similar analyses of lung DCs and lung CD8+ T cells [9], [13], [17].

First, to determine the functional ability of the lung CD4+ T cells to produce cytokines and chemokines, we isolated viable CD4+ T cells from the mechanically dispersed lung tissue of smokers with normal pulmonary function (*n* = 6) and COPD subjects (*n* = 23) **(Table S1)**, using immunomagnetic beads. After isolation, a median purity of 94% was obtained for the CD4+ fraction. Based on flow cytometry, these cells are predominantly CD3+ (92.7±4.8%), suggesting that contamination with DCs or macrophages was minimal. Representative histograms for CD4 staining are shown both before and after isolation **(Figs. S1A, S1B)**. These isolated CD4+ T cells were cultured without or with in vitro stimulation by plate-bound anti-CD3ε, and supernatants were analyzed by Luminex assay. Unstimulated lung CD4+ T cells made very little of any of the molecules measured and were typically below the minimum detectable limit of 5 pg/ml. with no relationship to lung function (data not shown).

**Figure 1:**
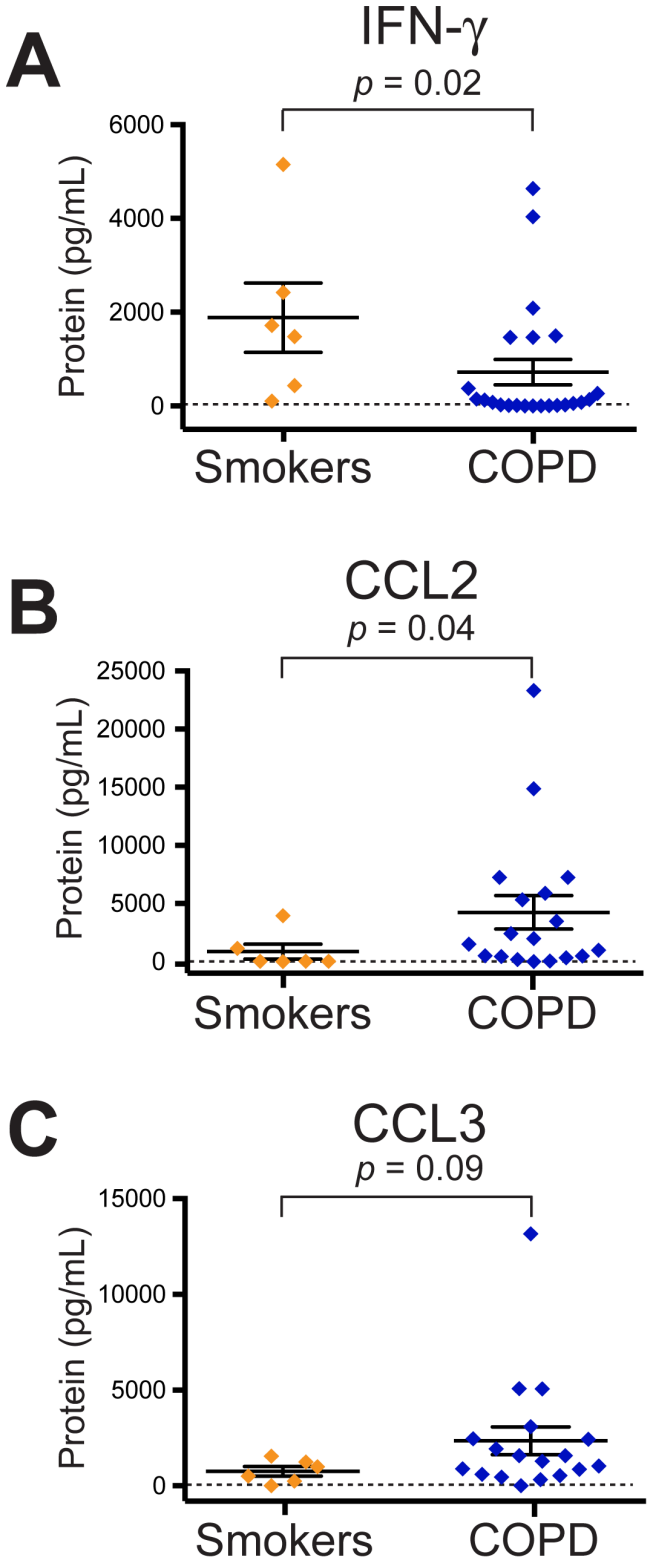
TCR-stimulated lung CD4+ T cells produce less IFN-γ protein but more CCL2 in COPD. Isolated lung CD4+ T cells were stimulated with plate-bound anti-CD3ε for 48 hours. Supernatants were collected and Luminex assays were used to measure (A) IFN-γ, (B) CCL2, and (C) CCL3 for smokers with normal spirometry (orange diamonds; *n* = 6) and COPD subjects (blue diamonds; *n* = 23 for panel A; *n* = 18 for panels B & C). Each symbol represents an individual patient; solid lines represent the mean ± SEM. The dashed line represents the minimum detectable concentration. The Mann Whitney t-test was used to determine significant differences between groups.

Stimulated lung CD4+ T cells from COPD subjects displayed significantly impaired IFN-γ production compared to the smokers with preserved pulmonary function **(** **Fig. 1A** **)**. Conversely, production of CCL2 was significantly augmented in the COPD subjects, and CCL3 showed a trend towards increased production, which did not attain statistical significance **(** **Fig. 1B, 1C** **)**. The significant differences in IFN-γ and CCL2 between subjects with COPD and smokers without COPD persisted after adjustment for sex, age, ICS use and pack-years. Interestingly, for all three analytes, a bimodal distribution can be noted in COPD subjects (i.e., cells of some subjects produced protein whereas a substantial fraction produced virtually none). There was no significant relationship between the production of IFN-γ and CCL2 or CCL3 by individual subjects (not shown). Following TCR stimulation, protein levels for the following analytes did not differ between COPD subjects and smokers without COPD, although concentrations (mean ± SD) were low in both groups: IL-10 (57±90 vs. 50±73 pg/mL); IL-13 (47±94 vs. 28±55 pg/mL); IL-17A (undetectable; minimum detectable concentration = 20 pg/mL); TNF-α (13±15 vs. 31±53 pg/mL); CCL5 (431±511 vs. 513±673 pg/mL).

Thus, global stimulation of CD4+ lung T cells of most subjects with COPD did not induce a T_H_1 cytokine profile, as that of the same cell type in many smokers with normal spirometry. Moreover, these results for lung CD4+ T cells were in marked contrast to the production of IFN-γ and TNF-α by lung CD8+ cells following the same TCR stimulation in subjects with COPD [9]. In additional experiments comparing TCR-stimulated CD4+ T cells and CD8+ T cells from the same 6 subjects, we are able to demonstrate a significant difference in the production of IFN-γ (88±144 pg/mL by CD4+ T cells vs. 377±399 pg/mL by CD8+ T cells; p = 0.03). Nevertheless, the lung CD4+ T cells of many COPD subjects did abundantly produce CCL2 and CCL3, indicating that they were not refractory to stimulation.

### Reduced T_EM_ Phenotype among Lung CD4+ T cells in COPD

**Figure 2:**
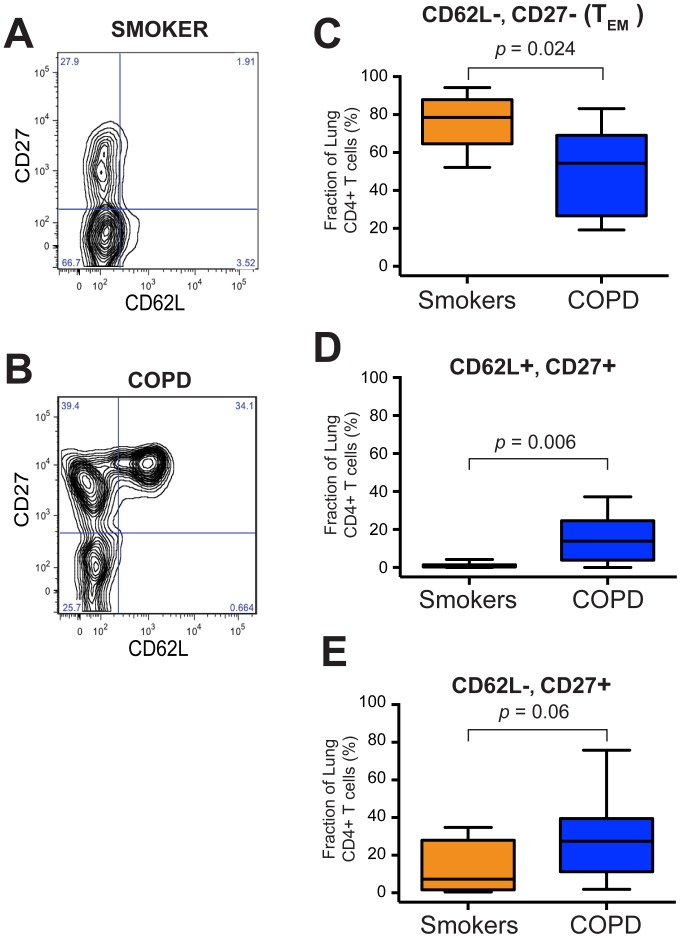
The T_EM_ subset of lung CD4+ T cells is significantly decreased in COPD patients. Single cell suspensions of mechanically disaggregated human lung tissue were stained and analyzed by flow cytometry. A, B. Representative flow plots showing staining for CD62L (horizontal axis) versus CD27 (vertical axis) on gated CD4+ T cells from (A) a smoker normal spirometry and (B) a subject with COPD. Small blue numbers are the percentage of CD4+ T cells in each quadrant. C–E. Aggregated percentages of lung CD4+ T cells from smokers with normal spirometry (orange; *n = *7) and COPD subjects (blue; *n* = 15) that were (C) negative for both CD62L & CD27 (T_EM_), (D) positive for both CD62L & CD27, or (E) negative for CD62L but positive for CD27. Box-&-whisker plots indicating the first & third quartiles (box), median (band) and range (whiskers). The Mann Whitney t-test was used to determine significance.

Next, to assess the phenotype of lung CD4+ T cells, we performed flow cytometry on single-cell suspensions from whole lung tissue **(Table S2)**. We measured surface expression of CD62L (a homing molecule also known as L-selectin) and CD27 (the receptor for the TNF family member CD70; down-regulated following T cell activation) **(** **Fig. 2A, 2B** **)**. Both these receptors are low-to-absent on effector memory T cells (T_EM_), which we have previously shown comprise the predominant subset among lung CD8+ T cells in all spirometrically-defined stages of COPD [9]. We used additional surface markers to gate on isolated lung CD4+ T cells, which were defined as being viable cells positive for CD45, CD3 and CD4, and negative for CD8 and CD56.

Compared to lung CD4+ T cells of smokers with preserved lung function (*n* = 7), those of subjects with COPD (*n* = 15), showed a significant decrease in the percentage of lung CD4+ T cells that were double-negative for CD62L and CD27 **(** **Fig. 2C** **)**, which have conventionally been thought to be the active T_EM_ phenotype responsible for most cytokine secretion. Importantly, however, as it was in all smokers with normal spirometry **(Figure S2A)**, T_EM_ remained the most frequent phenotype in the majority of COPD subjects (10 of 15) **(Figure S2B)**.

The decrease in the proportion of T_EM_ lung CD4+ T cells was accompanied by a significant increased frequency of cells double-positive for CD62L and CD27 **(** **Fig. 2D** **)**. This heterogeneous population could include both naïve T cells and central-memory T cell (T_CM_), i.e., exactly the mixture that would be anticipated to be found in organized lymphoid tissue, which is known to develop within lung parenchyma as COPD progresses [5]. Because these experiments did not include additional markers of memory phenotype (e.g., CD45RA, CCR7 or CD44), it is not possible to determine the percentage of naïve versus T_CM_ cells in individual subjects. Nevertheless, this double-positive fraction exceeded the percentage of T_EM_ cells among lung CD4+ T cells in only two of the 15 COPD subjects. Hence, it appears unlikely that the failure of lung CD4+ T cells to produce IFN-γ in response to plate-bound anti-CD3ε in the COPD subjects (Fig. 1) resulted from a predominance of naïve T cells. There was also a trend towards an increase in cells that were positive for CD27 but negative for CD62L **(** **Fig. 2E** **)**, which did not attain statistical significance. This phenotype is less well studied than T_EM_ in both humans and mice, but has been proposed to define recently activated CD4+ T cells in transition to the full effector phenotype [24].

No differences were observed in the frequency of CD4+ T cells as a percentage of leukocytes or of all lymphocytes when comparing smokers with preserved lung function (defined spirometrically) to COPD subjects (data not shown).

### Stimulated IFN-γ Production Correlates with the Percentage of Lung CD4+ T cells Expressing CD103+

**Figure 3:**
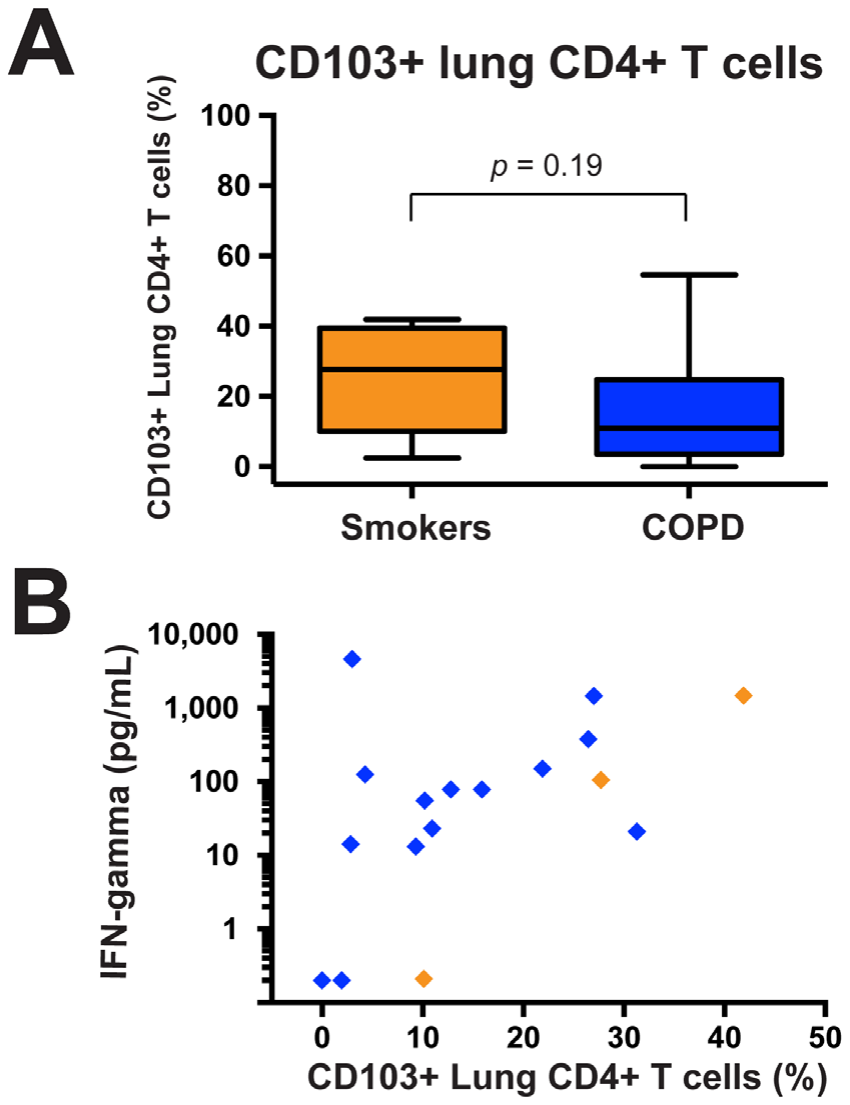
Production of IFN-γ protein by stimulated lung CD4+ T cells correlates with CD103+ expression. Isolated lung CD4+ T cells were stimulated with plate-bound anti-CD3ε for 48 hours. Supernatants were collected and Luminex assays were used to measure IFN-γ. A. Aggregated percentages of CD103+ cells among lung CD4+ T cells of smokers with normal spirometry (maize; *n* = 7) and COPD subjects (blue; *n* = 17). The Mann Whitney t-test was used to determine significant differences between groups. B. Correlation between stimulated IFN-γ protein concentrations (vertical axis, log scale) and percentage of CD103+ lung CD4+ T cells (horizontal axis). Smokers with normal spirometry (orange diamond; *n* = 3) and COPD subjects (blue diamonds; *n = *14) are a subset of those in panel A, as not all subjects had corresponding protein data. Statistics by Spearman correlation.

Until recently, T_EM_ and T_CM_ were the only defined memory T cell populations. However, a third memory population, tissue-resident memory T cell (T_RM_), has been identified in several tissues in murine models, especially in the lungs following resolution of viral respiratory infections [25], [26], and also in the healthy human lung [27]. We took advantage of the inclusion in these experiments of antibody against CD103 (alpha E integrin), one of the defining characteristics of this population. We found no difference between smokers with normal spirometry and COPD subjects **(Table S3)** in the fraction of cells expressing CD103 **(** **Fig. 3A** **)**, which were a minority of lung CD4+ T cells in both groups.

Interestingly, however, when we combined stimulated protein and flow data from both smokers and COPD subjects (total *n* = 15) **(Table S3)**, there was a significant correlation (*r_S_* = 0.55, p = 0.025) between the percentage of CD103+ cells and stimulated production of IFN-γ in vitro by the lung CD4+ T cells of the same individuals **(** **Fig. 3B** **)**. IFN-γ production did not correlate with the percentage of T_EM_ cells, CD62L+ CD27+ double-positive cells, or CD27+ CD62L− single-positive cells in this group.

### COPD Subjects and Smokers with Preserved Lung Function Largely Segregate into Two Distinct Groups based on Basal RNA Transcripts

**Figure 4:**
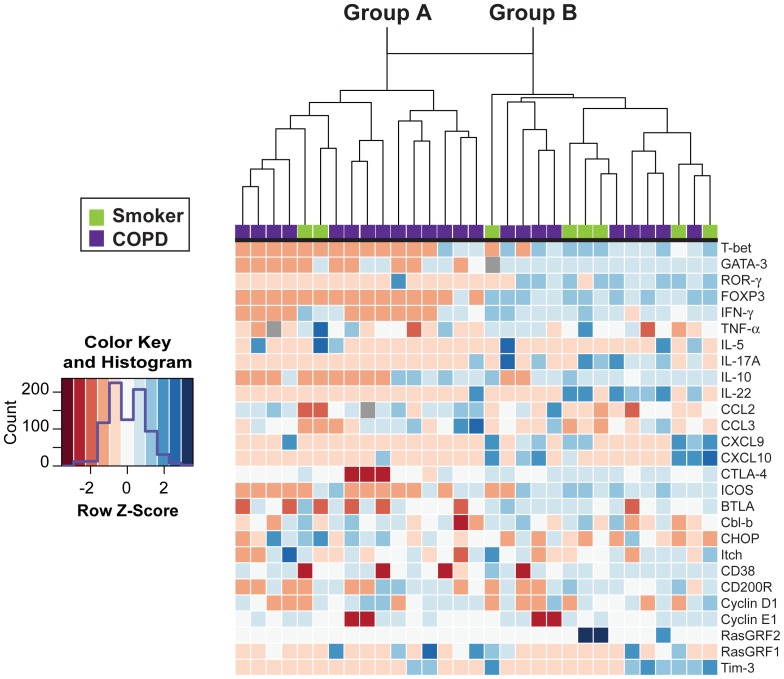
Unsupervised hierarchical clustering separates subjects based on unstimulated lung CD4+ T cell gene expression profiles. Transcripts for 27 genes were measured by real-time RT-PCR from isolated lung CD4+ T cells and results were displayed as a heat map. Columns represent individual subjects; gene identities are shown on the right. Red = down-regulated, white = unchanged, blue = up-regulated, grey = missing data. Based on spirometry plus clinical diagnosis, subjects were classified (top) either as smokers without COPD (green; *n* = 8) or COPD (purple; *n = *23). Unsupervised hierarchical clustering divided the subjects into two distinct groups, as indicated by the dendrogram. Group A contained 14 of 23 COPD subjects and two of eight smokers without COPD, while Group B consisted of the remaining six smokers without COPD and the other nine COPD subjects. The Row Z scores were calculated from log-transformed arbitrary units.

Next, we used real-time RT-PCR to analyze mRNA transcripts of 27 selected genes from isolated, unstimulated lung CD4+ T cells from smokers with preserved pulmonary function (*n* = 8) or COPD subjects (*n* = 23) **(Table S4)**. Candidate genes were chosen to include the transcription factors and target genes characteristic of the major polarized CD4+ T cells subsets; we also analyzed selected chemokines, regulatory receptors and genes associated with T cell anergy, in an attempt to understand the molecule basis of our earlier results. Data were log-transformed for heat map analysis. Interestingly, as illustrated by the dendrogram **(** **Fig. 4** **)**, unsupervised hierarchical clustering separated the entire group of subjects into two distinct groups. The group on the left (Group A) consisted of 14 of the 23 COPD subjects and two of the eight smokers with preserved lung function, while the group on the right (Group B) contained the remaining nine COPD subjects plus six smokers (**Fig. 4**). The division into two groups appeared to be driven by broad down-regulation, indicated by red to pink shading, of multiple polarizing transcription factors and their target genes in Group A subjects, which contrasted with the predominately upregulated expression (relative to reference genes, blue shading) of the same genes in Group B subjects.

**Figure 5:**
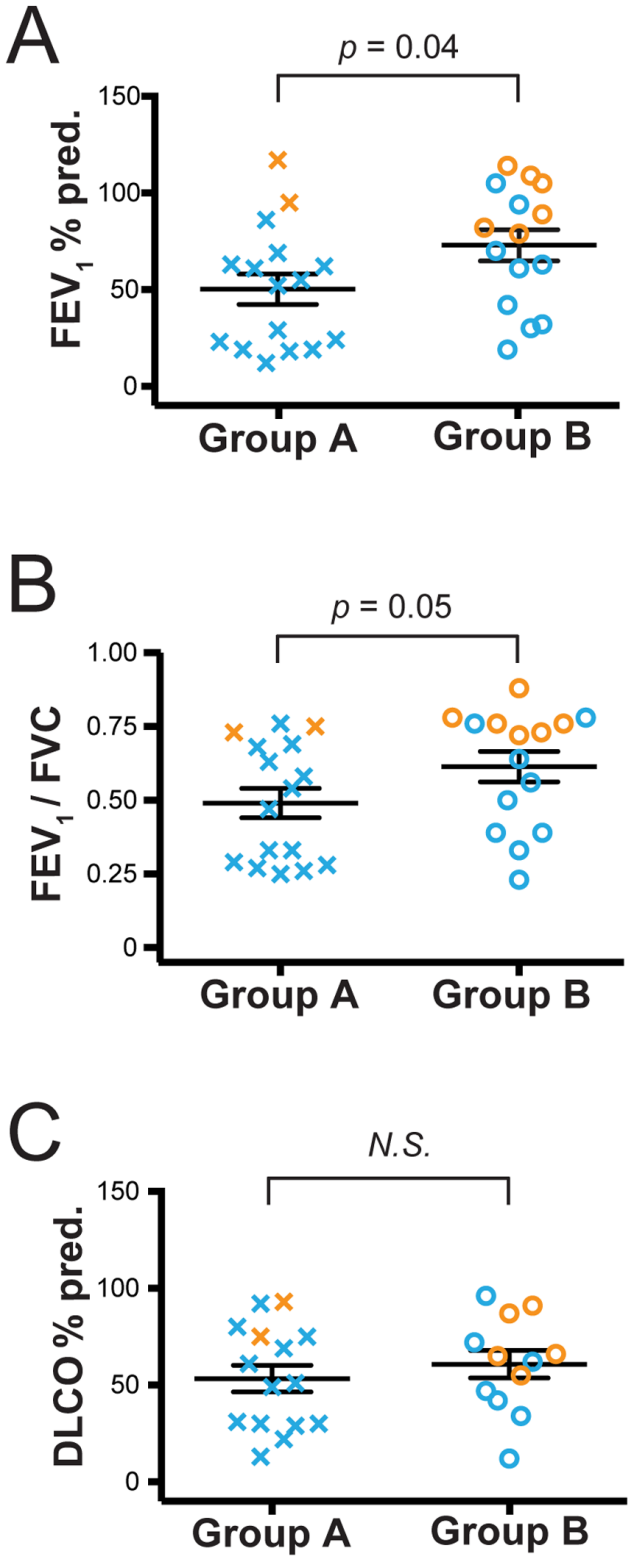
Group A subjects are characterized by worse spirometry than Group B subjects. Pulmonary function was assessed for all subjects and is presented based on the groups defined in Figure 4. (A) FEV_1%_ predicted, (B) FEV_1_/FVC ratio, and (C) DLCO % predicted are shown for Group A subjects (Xs; *n* = 16) and Group B subjects (circles; *n* = 15). Orange symbols represent smokers without COPD (*n* = 8) and blue symbols represent subjects with COPD (*n* = 23). Symbols represent individual patients, lines represent the mean ± SEM. The Mann Whitney t-test was used to determine significant differences between groups. N.S., not significant.

We next investigated whether the separation of subjects into Group A or Group B based on lung CD4+ T cell mRNA expression correlated with pulmonary function. Despite considerable overlap, we found that subjects in Group A had a significantly decreased FEV_1%_ predicted, as well as a lower FEV_1_/FVC ratio that just failed to attain significance, relative to all subjects in Group B (**Fig. 5A, 5B**). However, there was no difference in DLCO % predicted (**Fig. 5C**). We also found no difference between these transcript-defined groups when we analyzed age, sex, pack years, current smoking status, BMI, inhaled corticosteroid usage, or presence of malignancy or transplant as the indication for surgery **(** **Table 2** **)**.

**Table 2:** Comparison of clinical characteristics of Group A & Group B subjects^1^.

Group	A	B	*p* value
Subjects, n	16	15	
Sex ratio, M/F	8/8	11/4	0.27
Age, years (SD)	63 (8)	63 (11)	0.96
Smoking, pack-years (SD)	57 (20)	57 (53)	0.59
Smoking status (Active/Former^2^)	9/7	10/5	0.72
FEV1, % predicted (SD)	50 (31)	73 (31)	0.039
FEV1/FVC (SD)	0.49 (0.20)	0.61 (0.20)	0.05
Emphysema, % (SD)	23 (15)	24 (19)	0.98
Cancer as indication for surgery (yes/no)	9/7	11/4	0.46
Lung transplant (yes/no)	2/14	2/13	0.99
ICS 3 use (yes/no)	9/7	6/9	0.48

1 Data are presented as average (SD) except for sex ratios, smoking status, indication for surgery and ICS use; M, male; F, female;

2 former smoker defined as having quit for more than six months;

3 ICS, inhaled corticosteroids. The Mann Whitney t-test was used to determine significant differences between groups.

**Figure 6:**
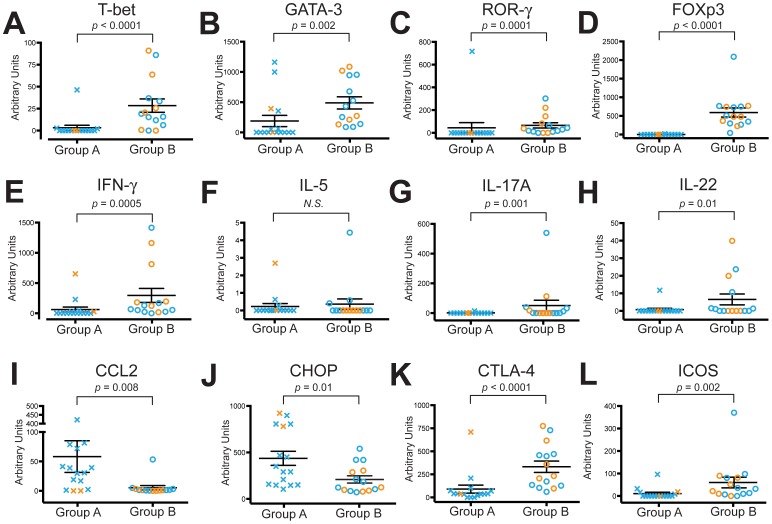
Group A subjects have significantly reduced transcripts for multiple polarizing transcription factors & signature cytokines. Lung CD4+ T cells were isolated for RNA analysis by real-time RT-PCR and mRNA transcripts are displayed as arbitrary units according to the subject groups defined by the heat map analysis in Figure 4. Group A (Xs; *n* = 16) and Group B (circles; *n* = 15). (A) T-bet, (B) GATA-3, (C) ROR-γ, (D), FOXP3, (E) IFN-γ, (F) IL-5, (G) IL-17A, (H) IL-22, (I) CCL2, (J) CHOP, (K) CTLA-4 and (L) ICOS. In both groups, orange symbols represent smokers without COPD (*n* = 8) and blue symbols represent subjects with COPD (*n* = 23). Symbols represent individual patients, lines represent the mean ± SEM. The Mann Whitney t-test was used to determine significant differences between groups. N.S., not significant.

Because hierarchical clustering examines levels of all 27 transcripts simultaneously, to determine which individual transcripts were actually significantly different between the two groups, we extended these analyses by performing Mann-Whitney t-tests while maintaining the Group A and Group B designations assigned by the dendrogram. Significant decreases were seen in the Group A subjects for the transcription factors for T_H_1, T_H_2, T_H_17, and T_Reg_ subsets, T-bet **(** **Fig. 6A** **)**, GATA-3 **(** **Fig. 6B** **)**, ROR-γ **(** **Fig. 6C** **)** and FOXP3 **(** **Fig. 6D** **)**, respectively, all of which were barely detectable in most Group A subjects. Transcripts for the T_H_1 signature gene IFN-γ **(** **Fig. 6E** **)** and for the T_H_17 cytokines IL-17A and IL-22 (**6G, 6H**) were also significantly reduced in Group A individuals, compared to Group B. The Th2 cytokine, IL-5, (**Fig. 6F**) showed no significant difference and transcripts were close to undetectable. IL-4 and IL-13 transcripts were undetectable, therefore they were not included in either the heat map or the individual analyses. Thus, in contrast to the balanced expression of transcription factors and genes for multiple polarized CD4+ T cell subsets in the Group B subjects, we found significantly decreased expression of all T cell subsets in Group A.

Importantly, however, we found elevated expression in lung CD4+ T cells from Group A subjects for two of the candidate genes. These were the chemokine CCL2 **(** **Fig. 6I** **)**, in agreement with the results of stimulated protein secretion in COPD subjects as a whole (Fig. 2), and CCAAT-enhancer-binding protein homologous protein (CHOP) **(** **Fig. 6J** **).** Thus, the virtually absent expression of mRNA transcripts for IFN-γ and multiple transcription factors seen in the Group A subjects does not reflect a global inability to elaborate any RNA transcripts.

### Examination of Possible causes for Reduced Polarization and Cytokine Transcripts in Group A Subjects

We analyzed other genes specifically to test several potential explanations for widespread gene down-regulation in Group A subjects. Significant reductions were seen in Group A in transcripts for three negative-regulatory members of the CD28 superfamily, CTLA-4, ICOS (**Fig. 6K, 6L**) and BTLA **(Fig. S3A)**, arguing against their involvement. Nor did we detect any significant differences in the E3 ubiquitin ligases ITCH and Cbl-b, which are reported to play a critical role in induction of T cell anergy (**Fig. S3B, S3C**) [28], [29]. No other significant differences were seen among the genes tested in **Figure 4**, which included the inhibitory molecule CD200R, CD38, cyclins D1 & E1, TIM3 or RasGRF1 & 2 (not shown).

Next, to test for senescence in lung CD4+ T cells of Group A individuals, we measured telomere length by a PCR-based method used recently to demonstrate that the peripheral blood leukocytes of COPD patients show reduced telomere length [4]. We found no significant differences between groups in the telomere repeat copy number to single-gene copy number (T/S) ratio (**Fig. S3D**), implying that the difference is not explained by global senescence of lung CD4+ T cells.

As mRNA yields permitted, we also measured expression by the isolated lung CD4+ T cells of additional genes in some subjects from these two groups (group A, *n* = 7, all with COPD; Group B, *n* = 9, 4 COPD, 5 smokers without COPD) **(Table S5)**. Although limited by the smaller sample size, for all genes in which there was a significant difference **(Table S6)**, expression was higher in Group B subjects, supporting the conclusions for the larger sample sizes in Figure 6. Importantly, Group A subjects showed significant reductions in three negative regulators of T cell function: Egr-2 and Egr-3 (which are upregulated by anergic T cells [30]). Together with the data in Figure 3 and Figures S3B & S3C, these results argue against anergy as a cause for the lack of polarization of lung CD4+ T cell in Group A subjects.

### IL-10 Transcripts Inversely Correlate with Emphysema Scores in Group A vs. Group B Subjects

**Figure 7:**
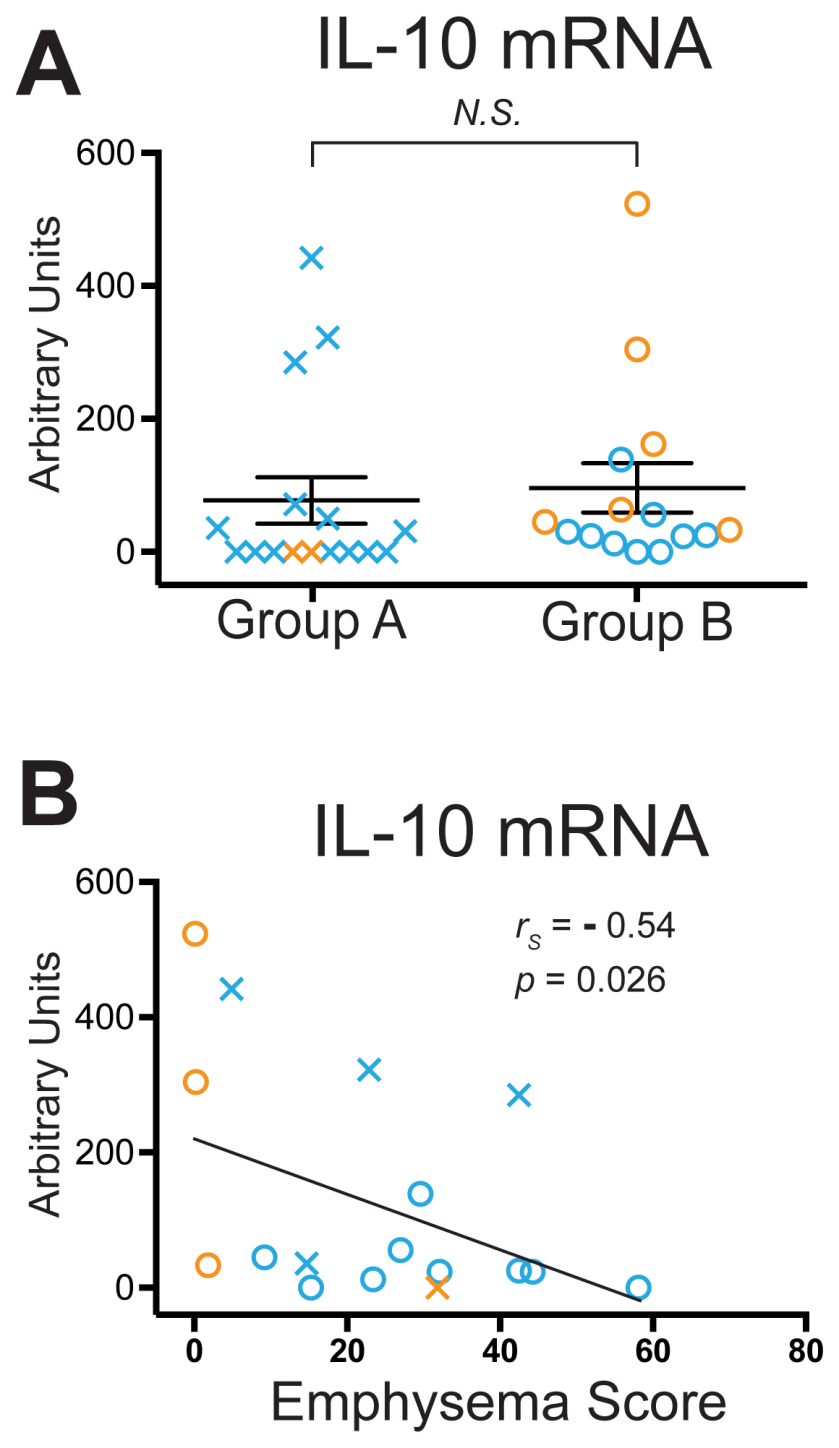
Decreased IL-10 mRNA transcripts in lung CD4+ T cells correlate with worsening emphysema. Lung CD4+ T cells were isolated for analysis of IL-10 mRNA by real-time RT-PCR. (A) mRNA transcripts presented in the Group A and Group B subject groups defined in Figure 4. Symbols, which represent individual subjects, are as described in the Legend to Figure 6; lines represent the mean ± SEM. The Mann Whitney t-test was used to determine significant differences between groups; N.S., not significant. (B) Relationship of IL-10 mRNA transcripts in lung CD4+ T cells, expressed as arbitrary units (vertical axis), to percent emphysema (−950 HU threshold) (horizontal axis) (*n* = 17). Spearman non-parametric analysis was used to calculate *r_S_* and *p* values.

We asked whether the impaired transcript expression of the lung CD4+ T cells in the Group A subjects might be associated with lung CD4+ T cell expression of IL-10, a potent immunomodulator capable of suppressing inflammatory cytokines in multiple cell types. We found no significant differences between the two groups in levels of IL-10 transcripts (**Fig. 7A**), or in IL-10 protein levels following stimulation of lung CD4+ T cells with plate-bound anti-CD3ε (data not shown). However, among the Group A and Group B subjects for whom analyzable (non-contrast) CT scans were available (*n* = 17), IL-10 transcripts did show a significant inverse correlation with emphysema score, as determined by non-parametric Spearman correlation (*r_S_* = −0.54; *p* = 0.026) (**Fig. 7B**). The direction and magnitude of this correlation was not changed after adjustment for ICS use, age, sex, or pack-years. No correlations of IFN-γ transcripts were seen with IL-10 or emphysema score.

### Group A Subjects have a Significant Reduction in the Frequency of CD62L−, CD27+ lung CD4+ T cells

Next, we used the flow cytometry data available on the Group A and Group B subjects to ask whether the marked disparity in lung CD4+ T cell polarization and expression of mRNA for multiple cytokine and chemokines correlated with expansion of T_CM_ cells or another well-defined T cell phenotype. Increased percentages of CD4+ T_CM_ and possibly of naïve CD4+ T cells in the lungs might be anticipated to accompany expansion of organized peribronchial lymphoid tissue, as has been shown to occur in more advanced COPD [5]. This was, in fact, what we found on analyzing subjects simply based on diagnosis of COPD (Fig. 2D). The data for this final analysis are a subset of those presented in Figure 1; however, because flow cytometry was necessarily performed within hours of tissue harvesting, we were not aware of which subjects comprised groups A & B until much later.

**Figure 8:**
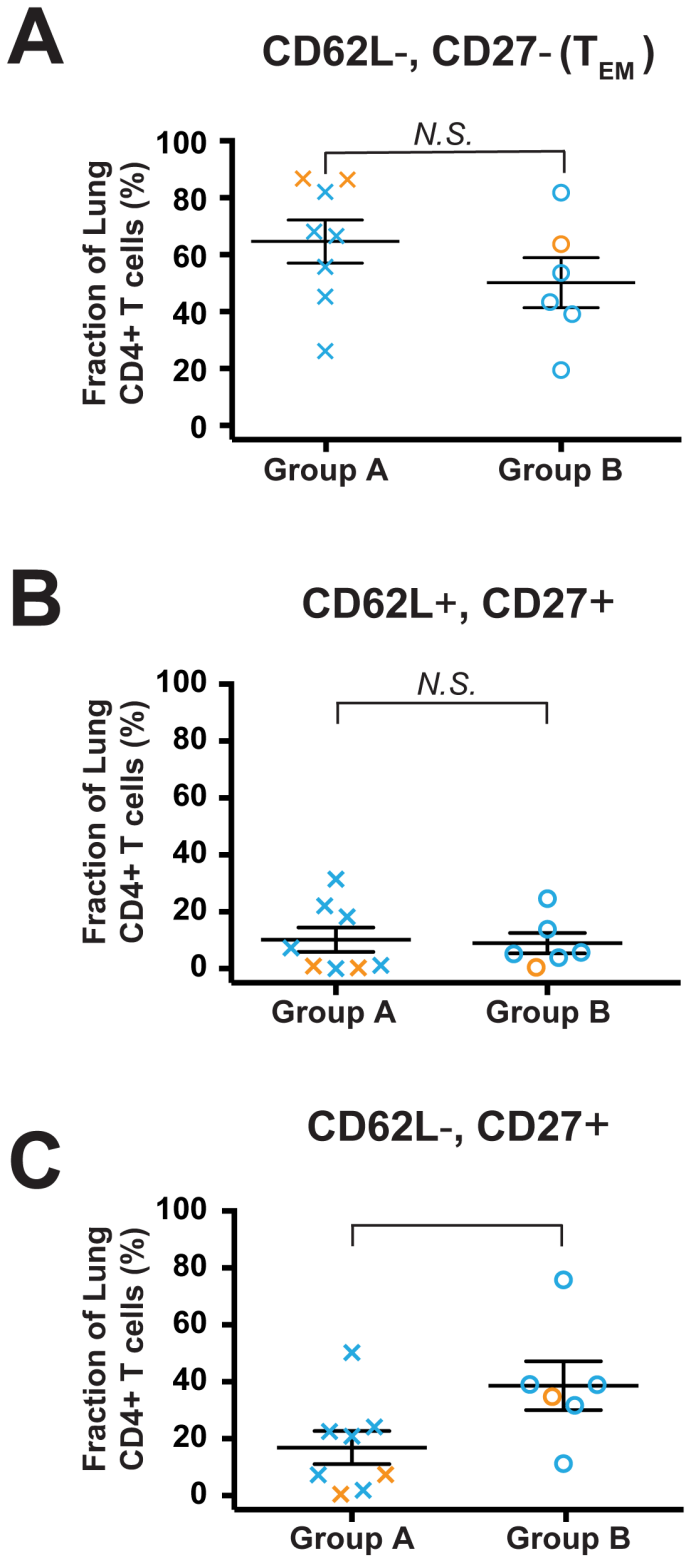
Group A subjects have expansion of an unusual population (CD62L−, CD27+), but not of T_EM_. Available flow cytometry data on subjects in Group A and Group B were analyzed to determine the fraction of lung CD4+ T cells in each of the quadrants defined by CD62L and CD27 expression. (A) CD62L− CD27− T_EM_; (B) CD62L+ CD27+; (C) CD62L− CD27+. Symbols represent individual patients, lines represent the mean ± SEM. Group A subjects (Xs; *n* = 8) and Group B subjects (circles; *n* = 6); orange symbols represent smokers without COPD (*n* = 3) and blue symbols represent subjects with COPD (*n* = 10). The Mann Whitney t-test was used to determine significant differences between groups. N.S., not significant.

There were no significant differences between Group A and Group B in the percentage of lung CD4+ T cells expressing either T_EM_ or CD62L, CD27 double-positive phenotypes **(** **Figs. 8A, 8B** **)**. Unexpectedly, there was a significant decrease in Group A subjects in the fraction of cells single-positive for CD27 **(** **Fig. 8C** **)**. Thus, we cannot readily relate the mRNA-defined Group A classification to a CD4+ T cell surface phenotype, although this analysis is obviously limited by the small number of subjects whose cells underwent both flow cytometric and quantitative real-time PCR analyses.

### Impaired Pulmonary Function can be Predicted by IFN-γ Transcript Levels

Finally, we returned to the entire group of COPD subjects and smokers without COPD on whom we had mRNA data (*n* = 31) to test for correlations of specific mRNA transcripts and FEV_1%_ predicted. Spearman non-parametric analysis found highly significant positive correlations with several analytes, including IFN-γ (*r* = 0.57, *p* = 0.0009), GATA-3 (*r* = 0.51, *p* = 0.004), CTLA-4 (*r* = 0.48; *p* = 0.006), FOXP3 (*r* = 0.37, *p* = 0.04), and an inverse correlation with CCL2 (*r* = −0.62, *p* = 0.0003) (data not shown).

To determine which of these variables were able to predict FEV_1%_ predicted independently, we performed multiple linear regression modeling. In a model incorporating age, sex, smoking history, ICS usage, BMI and DLCO % predicted, a higher FEV_1%_ predicted was associated with higher levels of IFN-γ transcripts (*p* = 0.04) **(** **Table 3** **)**. Higher FEV_1%_ predicted was also strongly associated with higher DLCO % predicted (*p* = 0.01). This statistical model implies that, unlike the strongly positive association we have previously described between advanced COPD stage and spontaneous [8] or stimulated [9] production of IFN-γ by lung CD8+ T cells, waning production of IFN-γ by lung CD4+ T cells is significantly associated with spirometrically-defined COPD progression.

**Table 3:** Linear regression model to evaluate ability of variables to predict FEV_1_% predicted.

Variable	B^1^	Std. error^1^	Sig.	95% CI Lower bound, Upper bound
Dependent variable = FEV_1_ (% predicted)				
Independent variables:				
Age	0.74	0.46	0.13	−0.23, 1.7
Sex	−2.7	9.3	0.78	−22.3, 16.9
Smoking, pack-years	−0.07	0.13	0.58	−0.34, 0.19
DLCO, % predicted	0.61	0.22	0.01	0.14, 1.1
Current smoking status	2.72	9.66	0.78	−17.6, 23.0
ICS	−20.3	11.7	0.10	−44.8, 4.3
BMI	−1.16	0.91	0.22	−3.1, 0.74
IFN-γ	0.03	0.02	0.04	0.00, 0.06

1 Unstandardized Coefficients. Sig., significance; CI, confidence interval.

## Discussion

Using multiple analytic techniques on viable lung CD4+ T cells isolated from 53 human subjects, this study disclosed five novel findings in COPD, relative to smokers with normal spirometry. First, following TCR stimulation, lung CD4+ T cells of most COPD subjects produced virtually no IFN-γ or other inflammatory cytokines, but many produced relatively more chemokines that could attract monocytes. Second, the surface phenotype of lung CD4+ T cells in COPD was less uniformly T_EM_ predominant. Third, basal gene expression in our entire group identified a phenotype, mainly in COPD subjects, characterized by markedly reduced mRNA for the transcription factors necessary to polarize multiple CD4+ T cell subsets (T_H_1, T_H_2, T_H_17 and FOXP3+ T_Reg_) and for their signature cytokines. This finding did not result from global inability to elaborate mRNA; greater expression of transcripts for inhibitory CD28 family members or for markers of anergy; or senescence (as indicted by reduced telomerase length). Subjects with this novel mRNA-defined phenotype (Group A) had significantly lower FEV1% predicted and non-significantly reduced FEV1/FVC ratios, but not DLCO, relative to subjects whose lung CD4+ T cells expressed a variety of transcripts. Fourth, considering both COPD and smoking subjects, transcripts for IL-10 correlated inversely with radiographically-defined emphysema extent, but not with spirometry. Fifth, multiple linear regression modeling identified expression of IFN-γ mRNA by unstimulated lung CD4+ T cells as an independent predictor of FEV1% predicted, suggesting that loss of T_H_1 polarization may contribute to, rather than result from, spirometrically-defined COPD progression. These findings provide new insights into how loss of appropriate immune functions mediated by lung CD4+ T cells might contribute to COPD pathogenesis in susceptible individuals.

Defending the lungs against diverse pathogens while minimizing lung inflammation requires multiple CD4+ T cell subsets. These subsets can be defined either by their polarized production of specific cytokines (e.g., T_H_1, T_H_2, etc.) or in an independent fashion, by their expression of cell surface receptors. We will first consider the implications of our findings for each of these two T cell definition schemes, before discussing their relationship to anticipated results from published data on lung CD8+ T cells, and our study’s limitations.

### Heterogeneity of Cytokine-defined Subsets of Lung T cells in COPD

Measurement of basal mRNA transcripts provide a snapshot of a given cell type’s activity at the time of collection. We found that basal levels of mRNA transcripts for IL-10 and IFN-γ in lung CD4+ T cells were unrelated in individual subjects, and were inversely correlated with emphysema extent vs. spirometry, respectively. These data did not depend on vitro experimental manipulation, and derived from regression analysis of all subjects with available data, making them the least subject to concerns about categorization by diagnosis. By providing novel molecular insights into the known clinical heterogeneity of this complex syndrome, we consider the correlation of specific basal cytokine transcripts in lung CD4+ T cells with distinct clinical phenotypes the most noteworthy of our current findings.

Based on our collective cytokine data, we propose that two largely independent types of lung CD4+ T cell dysfunction develop in some susceptible smokers and contribute to development of individually variable in COPD phenotypes: (1) deficient CD4+ T cell elaboration of IL-10, which fails to check auto-aggressive activities of lung CD8 and NK cells leading to emphysema; and (2) pervasive loss of CD4+ T cell polarization, which leads to non-emphysematous pathologies that cause airflow obstruction. The ultimate molecular bases for these dysfunctional lung CD4+ T cell phenotypes remain uncertain. Some recent evidence supports a systemic defect of CD4+ T cell polarization in COPD [31], but we currently favor a specific effect of the lung environment uniquely affecting lung CD4+ T cells for the loss of polarization (Group A) phenotype.

These contrasting associations between IL-10 and IFN-γ provide a clear example of the importance of strictly defining specific COPD phenotypes [32] for mechanistic studies. Although generally considered part of the overall COPD syndrome, increasing evidence implies that emphysema may result from pathogenic mechanisms distinct from those fostering airways disease. As recently stated by Manichaikul and colleagues “Emphysema on computed tomography (CT) is only moderately correlated with lung function, is absent in some patients with COPD, and occurs in the absence of COPD” [33]. The inverse association between IL-10 transcripts and emphysema extent we found is particularly significant given the independent role of emphysema in mortality [34].

IL-10 is a potently anti-inflammatory cytokine that can modulate many inflammatory and parenchymal cell types. Our IL-10 results support and extend a previous analysis of whole lung tissue [14], but contradict analysis of CD4+ T cells in bronchoalveolar lavage [35], which may indicate anatomic compartmentalization of the pulmonary immune response. A potential strength of our approach is that it derives from distal lung parenchyma, the chief site of pathology in COPD [36]. Although IL-10 can either stimulate or inhibit CD8+ T cells depending on the context [37], IL-10 does act on antigen-presenting cells to prevent their maturation and to decrease stimulatory molecule expression [38]. Hence, deficient Il-10 production by lung CD4+ T cells might indirectly permit greater activation of auto-aggressive CD8+ lung T cells. IL-10 polymorphisms have been associated with airflow obstruction (but not emphysema) in some [39], [40] but not all studies [41], suggesting a possible genetic basis.

Why basal IFN-γ transcripts in lung CD4+ cells significantly and independently correlate with abnormal spirometry is not immediately apparent, especially given the strong evidence that IFN-γ can drive COPD progression. Importantly, we acknowledge the possibility that IFN-γ is serving as a surrogate for some factor(s) not measured in our study but also defective in the Group A subjects. Although excessive CCL2 production could fuel recruitment to the lungs of monocytes that could specifically drive inflammation, CCL2 was not an independent predictor of spirometric outcomes in our logistic model.

Our finding that lung CD4+ T cells from most smokers with normal spirometry produced a variety of cytokines on stimulation agrees with a study of normal human lung tissue [27], despite difference in methods of isolation and stimulation. By studying both emphysematous and non-emphysematous lungs, our results complement and are not necessarily at odds with a study of lung CD4+ T cells from emphysematous subjects, which detected stimulated IFN-γ and IL-17A production using sensitive intracellular flow cytometric staining [15]. Because both those investigators and we examined relatively small groups of subjects, it is possible that our two laboratories have identified separate prototypic molecular subtypes within the broad spectrum of COPD phenotypes. Alternatively, an intriguing possibility that would reconcile results of our two studies would be an in vivo block in signaling just distal to the TCR, detectable by our approach but not by PMA/ionomycin stimulation, which activates downstream of TCR.

The finding of reduced FOXP3 transcripts in lung CD4+ T cells of many COPD subjects, which aligns with several studies on lung T_Reg_ cells in COPD [14], [27], [42], is significant given the conflicting data on this subset. Our conclusions disagree with two studies [43], [44] that relied on CD25 expression, a less specific indicator of T_Reg_ cells. Because those studies also analyzed bronchoalveolar lavage fluid, disparity from our data might also reflect the difference in anatomic compartmentalization. Differences in methodology complicate direct comparison with the results of a study that used immunohistology to determine that CD4+ FOXP3+ cells were increased in the follicles from COPD patients compared to smokers and non-smokers [45]. Once again, the range of this measurement in our individual COPD subjects argues that a deficiency in inducible T_Reg_ may not be uniform in this heterogeneous disease. Considerably larger numbers of COPD subjects must be analyzed before this or any deficit can be accepted as a universal feature of COPD pathogenesis.

### Cell Surface Receptor-defined Subsets of Lung T cells in COPD

Independently of polarized cytokine production, T cell subsets can also be categorized using surface receptors that correlate with activation history, trafficking and memory status. For over a decade [46], surface receptor-defined T cell categories have consisted of naïve cells, short-lived T_EM_ cells and persistent T_CM_ subsets. These three categories have been demonstrated most convincingly in CD8+ T cells. In this study, we identified T_EM_ using low expression of CD62L and CD27, a valid indicator for both CD8+ and CD4+ human subsets. Our identification of the T_EM_ subset as the majority of lung CD4+ T cells in smokers without COPD also agrees with results from normal human lung tissue [27], although we identified T_EM_ using different, but equally well-established receptors. That lung CD4+ T cells are predominately T_EM_ in COPD agrees with an earlier study [12]. Because T_EM_ have a lower activation threshold than naïve or T_CM_ cells, our flow cytometric data do not explain the reduced IFN-γ protein production by many of our COPD subjects. Our data also suggest that Group A subjects may be relatively deficient in T follicular helper cells, which strongly express ICOS and CXCR5, the receptor that guides homing of CD4+ T cells to germinal centers [47], [48]. Because CD4+ regulation is essential to prevent autoimmunity during immunoglobulin class-switching, our findings may partially explain detection of auto-antibodies in advanced COPD [14], [49], especially given the observation that CD40L appears reduced in Group A subjects (p = 0.055, **Table S4**).

Further, we provide novel insight on the recently described T resident-memory (T_RM_) subset [50]. Our data extend previous finding of a human lung T_RM_ population in normal subjects, by showing that the percentage CD4+ T cells expressing CD103, a marker of intraepithelial localization, correlates directly with basal transcripts for IFN-γ among both smokers and COPD subjects. Based on results in murine models, the CD4+ T_RM_ cells in the lungs of both smokers and COPD subjects likely result from previous viral respiratory infections [50].

However, assigning tissue-resident T cells to receptor-defined categories has recently become more difficult for human CD4+ T cells. Several receptors that reliably subdivide CD8+ T cells, including CD28 (which we measured) and CCR7 (which we did not), are now known to be expressed by both naïve and T_CM_ human CD4+ cells [50], [51]. Hence, because our flow cytometry experiments were originally designed to analyze CD8+ lung T cells in the same samples by eight-color analysis and did not include determination of CD45 isoforms, we cannot distinguish the naïve and T_CM_ CD4 subsets in the current data. It is intriguing that COPD subjects had increased percentages of a different subset of lung CD4+ cells, CD62L−, CD27+ cells, which in a murine model of pulmonary tuberculosis have been linked to defective IFN-γ production [52].

Because T_EM_ still predominated in most COPD subjects, we doubt that the relatively small changes in the various non-T_EM_ phenotypes in COPD subjects adequately explain impaired stimulated IFN-γ protein responses. None of the lung CD4+ T cell subsets defined by CD62L and CD27 correlated with IFN-γ protein (not shown). Nor could surface phenotypic differences explain the absence of lung CD4+ cell polarization in Group A subjects, in whom the CD62L−, CD27+ subset was relatively reduced and other subsets unchanged. Thus, flow cytometry cannot at present be used to identify smokers lacking robust lung CD4+ T cell polarization.

### Relationship of Current Findings to Lung CD8+ T cells in COPD

Results for both stimulated cytokine production (both mRNA and protein) and for basal gene expression were contrary to expectations from several previous studies of lung T cells, including our own [5], [13], [35], [53]. Those studies established that as spirometry worsens, lung CD8+ T cells develop a strictly Tc1 phenotype with increasing potential for cytolysis, and that numbers and apparent activation states of both lung CD8+ and CD4+ T cells increase in parallel. However, the current experiments used identical isolation techniques and in over half of cases the same lung specimens as our previous studies of other lung leukocyte types [8], [9], [13], [17]. Hence, we believe that artifact related to our method of T cell isolation is highly unlikely. Additionally, in paired experiments isolating lung T cells from the six subjects, we showed that lung CD8+ T cells make significantly more IFN-γ following TCR stimulation than lung CD4+ T cells.

There are several ways in which the current findings and published data on CD8+ T cells in COPD can be reconciled with the known dependence for optimal induction of CD8+ cytolytic responses on CD4+ T cell help, mediated largely via cytokines. First, CD8+ T cells might still receive adequate help in regional lymph nodes if defective production of inflammatory cytokines by CD4+ T cells in COPD were limited to the lungs, but that possibility has recently been questioned [31]. Additionally, CD4-independent CD8+ T cells responses have been observed in tumors and several infectious diseases [54]. NK cells can induce primary CD8+ T cell immunity against intracellular infections [55] in the absence of CD4+ T cells. Proliferation and differentiation of antigen-specific CD8+ T cells can also be stimulated directly by Type I IFNs [56], which multiple cell types can elaborate. CD8+ T cells are also typically subjected to negative control by FOXP3+ T_Reg_ cells [57], which our data suggest are reduced in many COPD subjects with the Group A phenotype.

### Limitations

This study has several limitations. Arguably the greatest is that not all studies could be performed on samples from all subjects, restricting the sizes of groups in which multiple variables could be compared. Hence, we welcome examination of the veracity of our conclusions in larger groups of subjects. Another is its dependence on tissue removed for clinical indications, raising possible questions of generalizability. Because our subjects were candidates for surgery, they likely have fewer co-morbidities and superior performance status for spirometrically-defined stage, relative to all COPD patients of similar age. Hence, it is unlikely that debility explains our findings. None of our subjects had suffered a respiratory infection within six weeks of surgery, and we strictly avoided any areas suspicious for possible post-obstructive pneumonia. Importantly, we found no correlations of our findings with malignancy as the indication for surgery or with use of ICS. Although 11 of our samples were explants harvested at the time of lung transplantation, recipients in our program do not receive pre- or intraoperative immunosuppression prior to lung removal. Additionally, we do not present data on co-stimulation of lung CD4+ T cells via CD28 or CD2 in addition to CD3, and therefore, do not claim that these cells are utterly refractory to optimal stimulation. Future studies will be needed to explore that point and to determine the ultimate molecular basis for our findings.

### Conclusion

In summary, we demonstrate that despite evidence of an activated phenotype, lung CD4+ T cells in many subjects with COPD are not polarized to any conventional subset and do not readily produce inflammatory cytokines when polyclonally stimulated. We identified significant independent correlations of levels of unstimulated mRNA transcripts in lung CD4+ T cells with specific COPD phenotypes: reduced IL-10 transcripts were associated with emphysema, whereas pervasive absence of T-cell polarization was associated with airflow obstruction. By delineating the sharp contrast with the behavior of other lung leukocyte subtypes in advanced disease, our data highlight the value of analyzing isolated cell types to disclose findings not evident from global analyses of gene expression. These findings imply that, at least in some subjects, advanced COPD may be a state of simultaneous local immune deficiency and dysregulation, a novel paradigm for this leading worldwide cause of death and disability.

## Supporting Information

Figure S1 **Representative staining for CD4 on isolated lung T cells.** A, B. Representative histograms showing staining for CD4 lung T cells before (A) and after (B) isolation by immuno-magnetic beads.
(TIF)

Figure S2 **Memory phenotypes of lung CD4+ T cells in individual subjects.** A, B. Percentages of lung CD4+ T cells in the four possible quadrants defined by analysis of CD62L & CD27. Data are stacked columns for each subject (numbers on horizontal axis); double-negative cells (T_EM_) are colored violet and located on the bottom, followed in ascending order by cells that are single positive for CD27 (neon green), double-positive (T_CM_ and naive) (scarlet), and single-positive for CD62L (mustard-colored).
(TIF)

Figure S3 **The Group A phenotype is not characterized by genes associated with anergy, negative co-stimulation or by senescence.** Lung CD4+ T cells were isolated either for (A–C) RNA analysis by real-time RT-PCR or (D) to harvest DNA for analysis of the ratio of numbers of telomere repeats (T) to number of a known single copy gene, using the comparative threshold cycle method of PCR analysis [19]. (A) BTLA, (B) ITCH, (C) Cbl-b are shown for Group A subjects (Xs; *n* = 16) and Group B subjects (circles; *n* = 15). Orange symbols represent smokers without COPD (*n* = 8) and blue symbols represent subjects with COPD (*n* = 23). Symbols represent individual patients, lines represent the mean ± SEM. The Mann Whitney t-test was used to determine significant differences between groups. N.S., not significant.
(TIF)

Table S1 **Summary of demographics, smoking history, spirometry, indication for surgery and ICS usage for subjects used in stimulated protein experiments.**
(DOCX)

Table S2 **Summary of clinical characteristics of subjects used in flow cytometry experiments.**
(DOCX)

Table S3 **Summary of clinical characteristics of subjects used in CD103 versus IFN-γ correlation experiments.**
(DOCX)

Table S4 **Summary of clinical characteristics of subjects used in mRNA heat map analysis.**
(DOCX)

Table S5 **Summary of clinical characteristics of subjects used to measure additional mRNA transcripts.**
(DOCX)

Table S6 **Human lung CD4 transcripts in Group A versus Group B subjects.**
(DOCX)
