# Supplementary figures and images for: Basal Gene Expression by Lung CD4+ T Cells in Chronic Obstructive Pulmonary Disease Identifies Independent Molecular Correlates of Airflow Obstruction and Emphysema Extent
Source: PLoS One. 2014 May 7;9(5):e96421. doi: 10.1371/journal.pone.0096421 (PMC4013040; doi:10.1371/journal.pone.0096421)

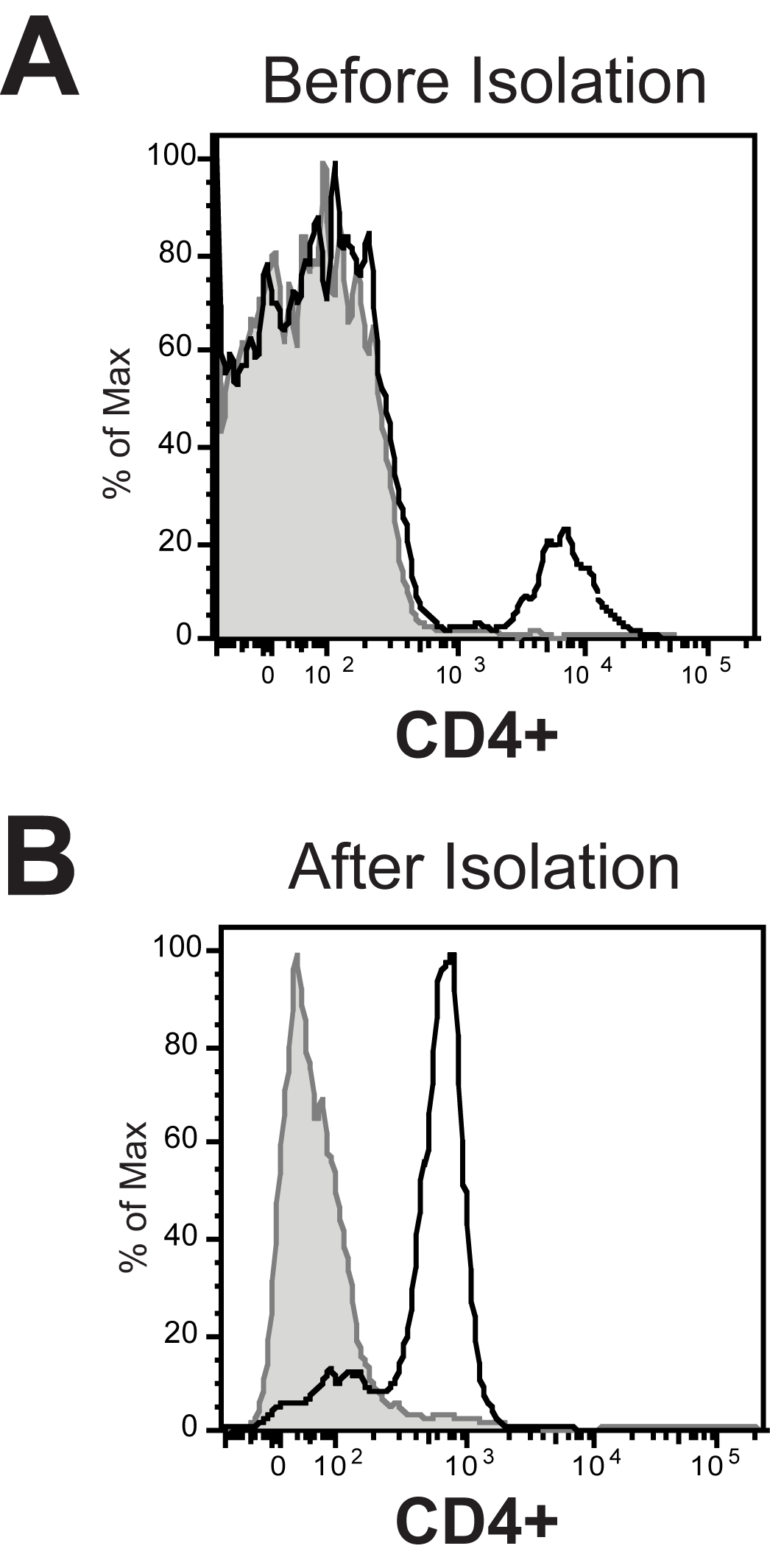

Supplement: Figure S1 — Representative staining for CD4 on isolated lung T cells. A, B. Representative histograms showing staining for CD4 lung T cells before (A) and after (B) isolation by immuno-magnetic beads. (TIF) [file pone.0096421.s001.tif]

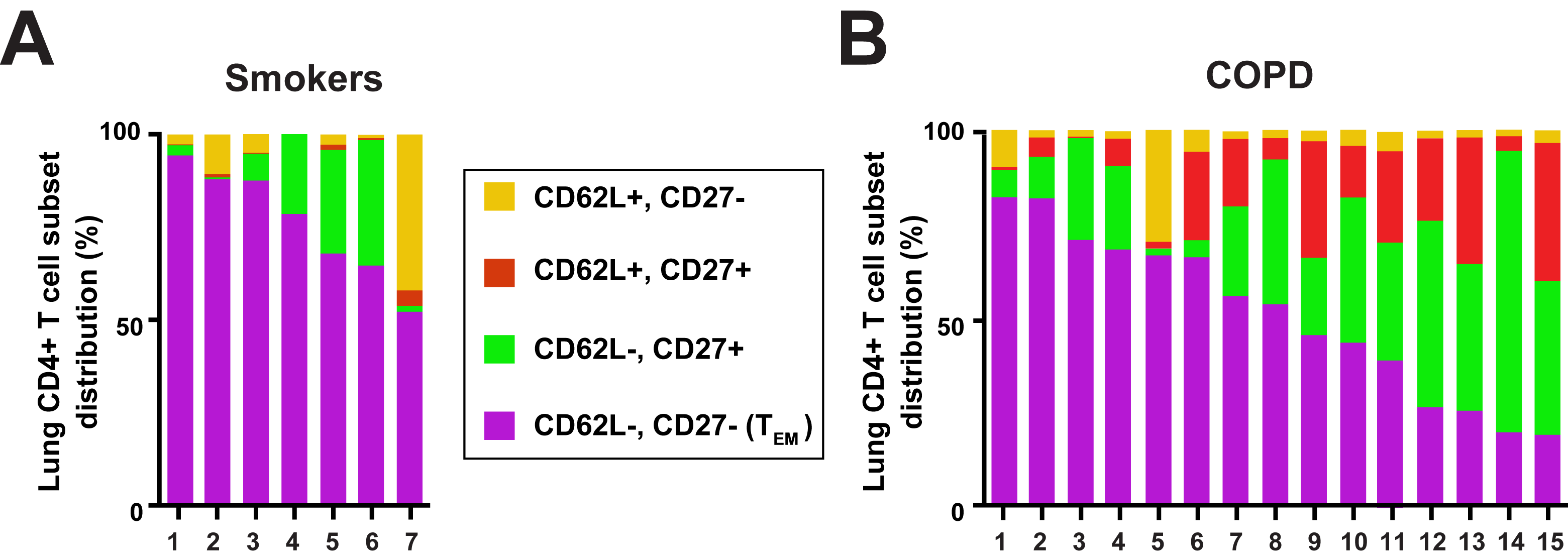

Supplement: Figure S2 — Memory phenotypes of lung CD4+ T cells in individual subjects. A, B. Percentages of lung CD4+ T cells in the four possible quadrants defined by analysis of CD62L & CD27. Data are stacked columns for each subject (numbers on horizontal axis); double-negative cells (TEM) are colored violet and located on the bottom, followed in ascending order by cells that are single positive for CD27 (neon green), double-positive (TCM and naive) (scarlet), and single-positive for CD62L (mustard-colored). (TIF) [file pone.0096421.s002.tif]

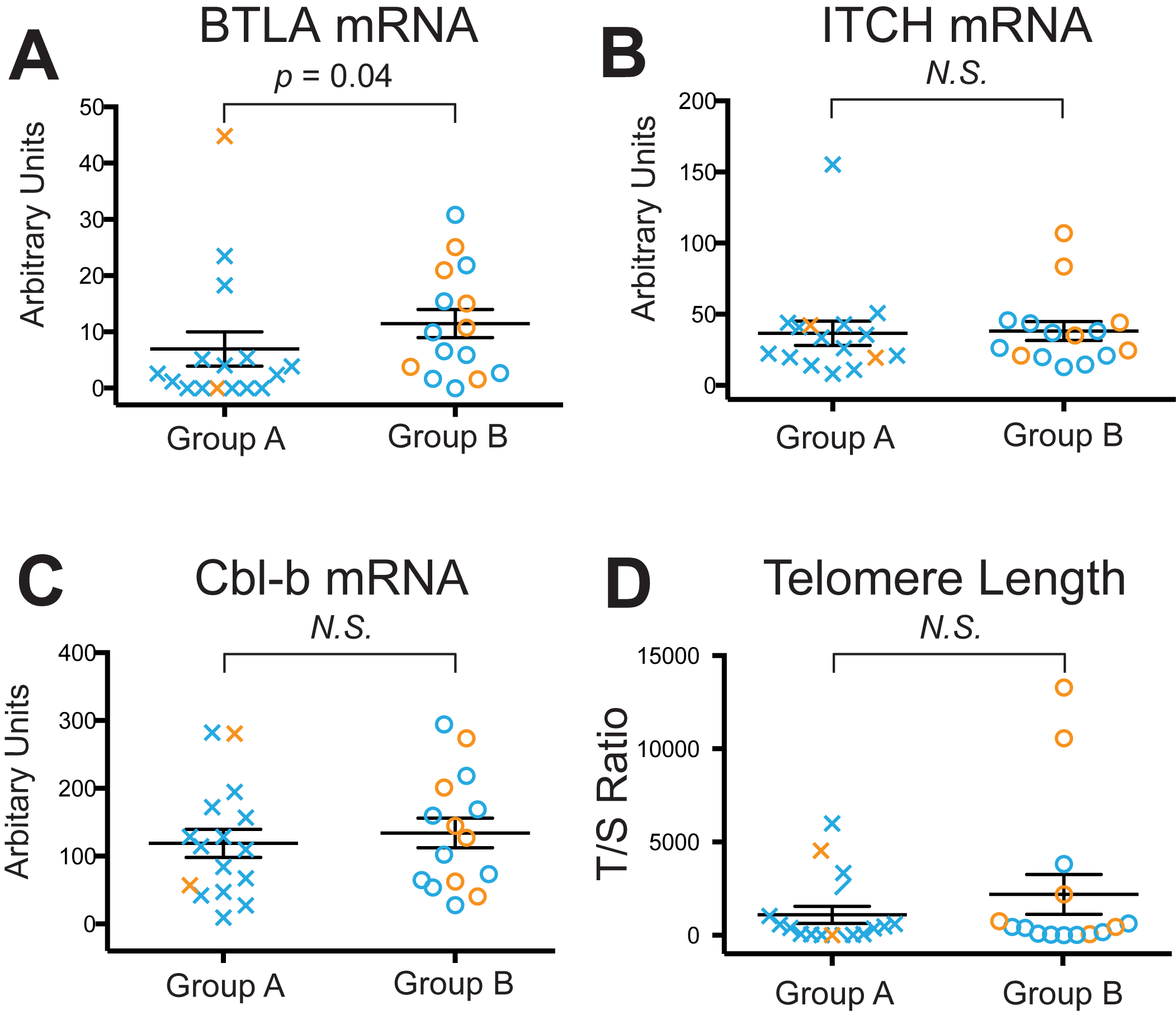

Supplement: Figure S3 — The Group A phenotype is not characterized by genes associated with anergy, negative co-stimulation or by senescence. Lung CD4+ T cells were isolated either for (A–C) RNA analysis by real-time RT-PCR or (D) to harvest DNA for analysis of the ratio of numbers of telomere repeats (T) to number of a known single copy gene, using the comparative threshold cycle method of PCR analysis [19]. (A) BTLA, (B) ITCH, (C) Cbl-b are shown for Group A subjects (Xs; n = 16) and Group B subjects (circles; n = 15). Orange symbols represent smokers without COPD (n = 8) and blue symbols represent subjects with COPD (n = 23). Symbols represent individual patients, lines represent the mean ± SEM. The Mann Whitney t-test was used to determine significant differences between groups. N.S., not significant. (TIF) [file pone.0096421.s003.tif]
