# Supplementary material for: Basal Gene Expression by Lung CD4+ T Cells in Chronic Obstructive Pulmonary Disease Identifies Independent Molecular Correlates of Airflow Obstruction and Emphysema Extent
Source: PLoS One. 2014 May 7;9(5):e96421. doi: 10.1371/journal.pone.0096421 (PMC4013040; doi:10.1371/journal.pone.0096421)
Supplement: Table S1 — Summary of demographics, smoking history, spirometry, indication for surgery and ICS usage for subjects used in stimulated protein experiments. (DOCX) [file pone.0096421.s004.docx]

**Table S1.**  **Summary of demographics, smoking history, spirometry, indication for surgery
and ICS usage for subjects used in stimulated protein experiments ^1^.**

| Group | Smokers with normal spirometry | COPD | *p* value |
| --- | --- | --- | --- |
| Subjects, n | 6 | 23 |  |
| Sex ratio, M/F | 5/1 | 17/6 | 0.99 |
| Age, years (SD) | 69 (7) | 62 (11) | 0.17 |
| Smoking, pack-years (SD) | 70 (57) | 70 (37) | 0.76 |
| Smoking status (Active/Former ^2^) | 2/4 | 13/10 | 0.38 |
| FEV1, % predicted (SD) | 100 (18) | 39 (25) | < 0.0001 |
| FEV1/FVC (SD) | 0.79 (0.05) | 0.41 (0.18) | < 0.0001 |
| Cancer as indication for surgery (yes/no) | 6/0 | 10/13 | 0.02 |
| Lung transplant (yes/no) | 0/6 | 9/14 | 0.14 |
| ICS ^3^ use (yes/no) | 0/6 | 15/8 | 0.006 |

^1^, Data are presented as average (SD) except for sex ratios, smoking status, indication for surgery and ICS use; M, male; F, female; ^2^, former smoker defined as having quit for more than six months; ^3^ ICS, inhaled corticosteroids. The Mann Whitney t-test was used to determine significant differences between groups.
