# Supplementary material for: Basal Gene Expression by Lung CD4+ T Cells in Chronic Obstructive Pulmonary Disease Identifies Independent Molecular Correlates of Airflow Obstruction and Emphysema Extent
Source: PLoS One. 2014 May 7;9(5):e96421. doi: 10.1371/journal.pone.0096421 (PMC4013040; doi:10.1371/journal.pone.0096421)
Supplement: Table S2 — Summary of clinical characteristics of subjects used in flow cytometry experiments. (DOCX) [file pone.0096421.s005.docx]

**Table S2. Summary of clinical characteristics of subjects used in flow cytometry experiments ^1^.**

| Group | Smokers with normal spirometry | COPD | *p* value |
| --- | --- | --- | --- |
| Subjects, n | 7 | 15 |  |
| Sex ratio, M/F | 5/2 | 8/7 | 0.65 |
| Age, years (SD) | 57 (9) | 63 (10) | 0.24 |
| Smoking, pack-years (SD) | 37 (25) | 66 (42) | 0.14 |
| Smoking status (Active/Former ^2^) | 5/2 | 9/6 | 0.67 |
| FEV1, % predicted (SD) | 99 (10) | 46 (27) | < 0.0001 |
| FEV1/FVC (SD) | 0.76 (0.05) | 0.47 (0.20) | 0.0015 |
| Cancer as indication for surgery (yes/no) | 7/0 | 8/7 | 0.05 |
| Lung transplant (yes/no) | 0/7 | 3/12 | 0.52 |
| ICS ^3^ use (yes/no) | 1/6 | 10/5 | 0.06 |

^1^, Data are presented as average (SD) except for sex ratios, smoking status, indication for surgery and ICS use; M, male; F, female; ^2^, former smoker defined as having quit for more than six months; ^3^ ICS, inhaled corticosteroids. The Mann Whitney t-test was used to determine significant differences between groups.
