# Supplementary material for: Basal Gene Expression by Lung CD4+ T Cells in Chronic Obstructive Pulmonary Disease Identifies Independent Molecular Correlates of Airflow Obstruction and Emphysema Extent
Source: PLoS One. 2014 May 7;9(5):e96421. doi: 10.1371/journal.pone.0096421 (PMC4013040; doi:10.1371/journal.pone.0096421)
Supplement: Table S3 — Summary of clinical characteristics of subjects used in CD103 versus IFN-γ correlation experiments. (DOCX) [file pone.0096421.s006.docx]

**Table S3. Summary of clinical characteristics of subjects used in CD103 versus IFN-**γ **correlation experiments ^1^.**

| Group | Smokers with normal spirometry | COPD | *p* value |
| --- | --- | --- | --- |
| Subjects, n | 3 | 12 |  |
| Sex ratio, M/F | 2/1 | 7/5 | 0.99 |
| Age, years (SD) | 56 (13) | 61 (10) | 0.71 |
| Smoking, pack-years (SD) | 59 (10) | 71 (43) | 0.82 |
| Smoking status (Active/Former ^2^) | 1/2 | 6/6 | 0.99 |
| FEV1, % predicted (SD) | 101 (14) | 41 (24) | 0.002 |
| FEV1/FVC (SD) | 0.74 (0.01) | 0.44 (0.18) | 0.026 |
| Cancer as indication for surgery (yes/no) | 3/0 | 6/6 | 0.23 |
| Lung transplant (yes/no) | 0/3 | 3/9 | 0.56 |
| ICS ^3^ use (yes/no) | 0/3 | 10/2 | 0.02 |

^1^, Data are presented as average (SD) except for sex ratios, smoking status, indication for surgery and ICS use; M, male; F, female; ^2^, former smoker defined as having quit for more than six months; ^3^ ICS, inhaled corticosteroids. The Mann Whitney t-test was used to determine significant differences between groups.
