# Supplementary material for: Basal Gene Expression by Lung CD4+ T Cells in Chronic Obstructive Pulmonary Disease Identifies Independent Molecular Correlates of Airflow Obstruction and Emphysema Extent
Source: PLoS One. 2014 May 7;9(5):e96421. doi: 10.1371/journal.pone.0096421 (PMC4013040; doi:10.1371/journal.pone.0096421)
Supplement: Table S4 — Summary of clinical characteristics of subjects used in mRNA heat map analysis. (DOCX) [file pone.0096421.s007.docx]

**Table S4. Summary of clinical characteristics of subjects used in mRNA heat map analysis ^1^.**

| Group | Smokers with normal spirometry | COPD | *p* value |
| --- | --- | --- | --- |
| Subjects, n | 8 | 23 |  |
| Sex ratio, M/F | 6/2 | 13/10 | 0.43 |
| Age, years (SD) | 63 (11) | 63 (9) | 0.90 |
| Smoking, pack-years (SD) | 33 (22) | 67 (38) | 0.01 |
| Smoking status (Active/Former ^2^) | 5/3 | 14/9 | 0.99 |
| FEV1, % predicted (SD) | 99 (15) | 48 (27) | < 0.0001 |
| FEV1/FVC (SD) | 0.76 (0.05) | 0.48 (0.19) | 0.0002 |
| Cancer as indication for surgery (yes/no) | 8/0 | 12/11 | 0.03 |
| Lung transplant (yes/no) | 0/8 | 5/18 | 0.29 |
| ICS ^3^ use (yes/no) | 0/8 | 15/8 | 0.002 |

^1^, Data are presented as average (SD) except for sex ratios, smoking status, indication for surgery and ICS use; M, male; F, female; ^2^, former smoker defined as having quit for more than six months; ^3^ ICS, inhaled corticosteroids. The Mann Whitney t-test was used to determine significant differences between groups.
