# Supplementary material for: Basal Gene Expression by Lung CD4+ T Cells in Chronic Obstructive Pulmonary Disease Identifies Independent Molecular Correlates of Airflow Obstruction and Emphysema Extent
Source: PLoS One. 2014 May 7;9(5):e96421. doi: 10.1371/journal.pone.0096421 (PMC4013040; doi:10.1371/journal.pone.0096421)
Supplement: Table S5 — Summary of clinical characteristics of subjects used to measure additional mRNA transcripts. (DOCX) [file pone.0096421.s008.docx]

**Table S5. Summary of clinical characteristics of subjects used to measure additional mRNA transcripts ^1^.**

| Group | A | B | *p* value |
| --- | --- | --- | --- |
| Subjects, n | 7 | 9 |  |
| Sex ratio, M/F | 3/4 | 7/2 | 0.30 |
| Age, years (SD) | 63 (7) | 66 (11) | 0.66 |
| Smoking, pack-years (SD) | 48 (8) | 42 (45) | 0.22 |
| Smoking status (Active/Former ^2^) | 4/3 | 6/3 | 0.99 |
| FEV1, % predicted (SD) | 51 (25) | 81 (27) | 0.039 |
| FEV1/FVC (SD) | 0.48 (0.18) | 0.67 (0.17) | 0.02 |
| Cancer as indication for surgery (yes/no) | 4/3 | 7/2 | 0.60 |
| Lung transplant (yes/no) | 1/6 | 1/8 | 0.99 |
| ICS use (yes/no) | 4/3 | 2/7 | 0.30 |

^1^, Data are presented as average (SD) except for sex ratios, smoking status, indication for surgery, and ICS use; M, male; F, female; ICS, inhaled corticosteroids; ^2^, former smoker defined as having quit for more than six months.
