# Supplementary material for: Basal Gene Expression by Lung CD4+ T Cells in Chronic Obstructive Pulmonary Disease Identifies Independent Molecular Correlates of Airflow Obstruction and Emphysema Extent
Source: PLoS One. 2014 May 7;9(5):e96421. doi: 10.1371/journal.pone.0096421 (PMC4013040; doi:10.1371/journal.pone.0096421)
Supplement: Table S6 — Human lung CD4 transcripts in Group A versus Group B subjects. (DOCX) [file pone.0096421.s009.docx]

**Table S6. Human lung CD4 transcripts in Group A vs. Group B subjects ^1^**

| **RNA transcript** | **p-value** |  | **RNA transcript** | **p-value** |  | **RNA transcript** | **p-value** |
| --- | --- | --- | --- | --- | --- | --- | --- |
| CD40L | 0.055 |  | CXCR5 | 0.18 |  | IL-12R β2 | 0.14 |
| CD57 | 0.11 |  | CXCR6 | 0.98 |  | IL-17F | 0.33 |
| CD73 | 0.45 |  | ***CX3CL1*** | ***0.007*** |  | ILT-2 | 0.63 |
| CD80 | 0.49 |  | DGK | 0.94 |  | Integrin α1 | 0.90 |
| CD161 | 0.16 |  | ***EGR-2*** | ***0.016*** |  | IL-12R β2 | 0.14 |
| ***CCL17*** | ***0.0002*** |  | ***EGR-3*** | ***0.005*** |  | IL-17F | 0.33 |
| CCL22 | 0.78 |  | ***CCR8*** | ***0.0012*** |  | Integrin α2 | 0.74 |
| CCR1 | 0.96 |  | CCR10 | 0.42 |  | KLRG1 | 0.81 |
| CCR2 | 0.11 |  | CXCR5 | 0.18 |  | LAX1 | 0.71 |
| ***CCR3*** | ***0.029*** |  | CXCR6 | 0.98 |  | Nedd4 | 0.39 |
| CCR5 | 0.25 |  | ***CX3CL1*** | ***0.007*** |  | NKG2D | 0.34 |
| ***CCR6*** | ***0.015*** |  | GITR | 0.14 |  | ***PD-1*** | ***0.023*** |
| CCR7 | 0.59 |  | GM-CSF | 0.23 |  | PD-2 | 0.52 |
| ***CCR8*** | ***0.0012*** |  | GRAIL | 0.15 |  | PDL1 | 0.14 |
| CCR10 | 0.42 |  | ***Granzyme B*** | ***0.048*** |  | PDL2 | 0.29 |
| ***CXCL11*** | ***0.0007*** |  | IL-6 | 0.74 |  | ***Perforin*** | ***0.002*** |
| CXCL13 | 0.59 |  | IL-7R | 0.21 |  | TCR-ζ | 0.45 |
| CXCR3 | 0.53 |  | IL-12R β1 | 0.055 |  | ZEB-2 | 0.52 |

^1^, significant difference between Group A and Group B (Mann Whitney non-parametric t-test) are indicated in bold italics; in all cases in which there was a significant difference between groups, expression was greater for Group B subjects.
